# Mechanisms of sympathetic regulation during Apnea

**DOI:** 10.14814/phy2.13991

**Published:** 2019-01-28

**Authors:** Stephen A. Busch, Christina D. Bruce, Rachel J. Skow, Jaime R. Pfoh, Trevor A. Day, Margie H. Davenport, Craig D. Steinback

**Affiliations:** ^1^ Neurovascular Health Laboratory Faculty of Kinesiology, Sport, and Recreation University of Alberta Edmonton Alberta Canada; ^2^ Department of Biology Faculty of Science and Technology Mount Royal University Calgary Alberta Canada

**Keywords:** Breath‐holding, chemoreflex, hypoxia, sympathetic activity

## Abstract

Volitional Apnea produces a robust peak sympathetic response through several interacting mechanisms. However, the specific contribution of each mechanism has not been elucidated. Muscle sympathetic activity was collected in participants (*n* = 10; 24 ± 3 years) that performed four maximal volitional apneas aimed at isolating lung‐stretch (mechanical) and chemoreflex drive: (Ainslie and Duffin 2009) end‐expiratory breath‐hold, (Ainslie et al. 2013) end‐inspiratory breath‐hold, (Alpher et al. 1986) prehyperventilation breath‐hold, and (Andersson and Schagatay 1998) prehyperoxia breath‐hold. A final repeated rebreathe breath‐hold protocol was performed to measure the peak sympathetic response during successive breath‐holds at increasing chemoreflex stress. Finally, the influence of dynamic ventilation was assessed through asphyxic rebreathe. Muscle sympathetic activity was calculated as the change in burst frequency (burst/min), burst incidence (burst/100 heart‐beats), and amplitude (au) between baseline and prevolitional breakpoint. Rebreathe was analyzed at similar chemoreflex stress as inspiratory breath‐hold. All maneuvers increased muscle sympathetic activity compared to baseline (*P* < 0.01). However, prehyperoxia exhibited a smaller increase (+22.18 ± 9.13 burst/min; +25.52 ± 11.7 burst/100 heart‐beats) compared to inspiratory, expiratory, and prehyperventilation breath‐holds. At similar chemoreflex strain, rebreathe sympathetic activity was blunted compared to inspiratory breath‐hold (*P* < 0.01). Finally, muscle sympathetic activity was not different between the repeated rebreathe trials, despite elevated chemoreflex stress and lower breath‐hold duration with each subsequent breath‐hold. We have demonstrated an obligatory role of the peripheral, but not central, chemoreflex (prehyperventilation vs. prehyperoxia) in producing peak sympathetic responses. At similar chemoreflex stresses the act of dynamic ventilation, but not static lung stretch per se, blunts muscle sympathetic activity. Finally, similar peak sympathetic responses during successive repeated breath‐holds suggest a sympathetic ceiling may exist.

## Introduction

The performance of volitional breath‐holding (BH) is a unique physiological challenge integral to many activities. Individuals who perform maximal voluntary BHs, whether for recreational or occupational purposes, are subjected to dynamic stress that requires considerable voluntary control in order to overcome the unconscious urge to breathe. A maximal volitional BH can be characterized by two distinct phases: (1) the initial period where an individual easily suppresses the drive‐to‐breathe, and (2) the onset of involuntary breathing movements (physiological break‐point) where the drive‐to‐breath is no longer suppressed and diaphragmatic contractions unconsciously occur. Both phases occur prior to the resumption of breathing (volitional break‐point), where the individual is no longer able to volitionally maintain a closed airway (Parkes 2006; Skow et al. 2015).

Though maximal BH duration is highly variable between individuals (Parkes 2006; Heusser et al. 2009), the “struggle phase” prior to volitional break‐point is often shorter than the true physiological breakpoint. Alongside a heightened sensation to breathe, the struggle phase also exhibits considerable potentiation of efferent muscle sympathetic nerve activity (MSNA) (Hardy et al. 1994; Macefield and Wallin 1995a; Heusser et al. 2009; Breskovic et al. 2010). This heightened MSNA response results in a large pressor response that facilitates the redistribution of blood flow to vital organs (Andersson and Schagatay 1998; Andersson et al. 2002; Bain et al. 2018). Although the exact mechanisms that control MSNA are unclear, the peak MSNA response is proposed to be a net result of excitatory and inhibitory reflexes that change across both the initial and postphysiological breakpoint periods (Macefield and Wallin 1995b; Parkes 2006; Heusser et al. 2010; Steinback et al. 2010a,b). Shorter duration BHs see an initial change in MSNA through interactions between lung volume and cardiopulmonary baroreflex activation (Macefield and Wallin 1995b; Heusser et al. 2009), while the peak MSNA response during long duration BHs are believed to be driven primarily through progressive heightened chemical drive (hypoxia and hypercapnia) (Hardy et al. 1994; Leuenberger et al. 2001; Heusser et al. 2009). It is believed that through activation of both the carbon dioxide/pH sensitive central chemoreceptors (Somers et al. 1989a; de Burgh Daly 1997) and oxygen sensitive peripheral chemoreceptors (Hardy et al. 1994; Morgan et al. 1995; Seitz et al. 2013), and removal of afferent pulmonary/chest wall reflexes (Macefield and Wallin 1995a; Steinback et al. 2010b) that the peak MSNA response occurs immediately prior to volitional breakpoint. However, the exact roles of both chemical activation and pulmonary stretch inhibition on generating a peak MSNA response are unclear.

Though previous studies have attempted to identify mechanisms that contribute to MSNA augmentation during breath‐holding (Leuenberger et al. 2001; Khayat et al. 2004), and the cessation of MSNA immediately following breakpoint (Hardy et al. 1994; Seitz et al. 2013), it is still uncertain how differences in chemical and lung stretch affect the peak MSNA response itself. Therefore, the purpose of this study was to determine the mechanistic contribution of mechanical (lung stretch) and chemoreflex (hypoxia and hypercapnia) mechanisms regulating the peak MSNA response during a maximal BH. This was assessed through a several BHs that removed either peripheral or central chemoreflex activation. We also assessed the contribution of lung stretch on peak MSNA potentiation by loading and unloading the pulmonary stretch receptors. Finally, as MSNA activity is tightly coupled with the respiratory cycle (Seals et al. 1990), we compared the static versus dynamic lung stretch on MSNA augmentation at similar degrees of chemoreflex activation. We hypothesized that each BH maneuver would evoke varying peak MSNA responses prior to volitional breakpoint due to differences in chemoreflex activation and pulmonary stretch inhibition.

## Materials and Methods

This study received approval from the University of Alberta‐ Human (Biomedical) Research Ethics Board (Pro00048741, June 26th, 2014) and complies with the *Declaration of Helsinki*. Informed consent was obtained prior to all testing. Baseline demographics, cardiovascular characteristics, and the heart rate responses to two of the outlined protocols below (rebreathe and end‐inspiratory breath‐holding) have been previously reported in an investigation of cerebrovascular reactivity (Bruce et al. 2016). However, this study focuses on novel data related to sympathetic reactivity between several different BH and rebreathing maneuvers.

### Study participants

Ten healthy participants (males = 4; 24 ± 3 years) were recruited. All participants were nonsmokers and screened for normal respiratory, cardiovascular, or neurological function. In addition, none of the participants had previous experience or professional training with maximal voluntary BHs. Both female and male participants were pooled together as four of the six females were either using an IUD, or were on oral contraception. In addition, the two female participants not using oral contraception or an IUD were not considered outliers following data analysis. Participants avoided caffeine, physical activity, and other sympathetic stimulants for 12 h prior to testing.

### Instrumentation

Participants voided their bladder and were then seated in a semirecumbent position prior to instrumentation and commencement of protocol. Cardiovascular instrumentation included recording of ECG (Lead II) and arterial blood pressure (finger photoplethysmography; Finometer Pro, Finapres Medical Systems, Netherlands). Both measures were collected continuously at 1000 Hz (ADInstruments, Chart Pro v8.3.1). Beat‐by‐beat mean, systolic and diastolic arterial pressures (MAP; SBP; DBP), were calculated from the continuous arterial pressure waveform. Beat‐by‐beat cardiac output (CO) was calculated using the Model Flow algorithm (Finometer Pro, Finapres Medical Systems, Netherlands) and used to calculate total peripheral resistance (TPR = MAP/CO). SpO2 was continually assessed (pulse oximetry; Nellcor; Medtronic, Ireland). Spirometry flow (L/sec) and volume (L) was assessed via pneumotachometer (Hans Rudolph Inc., MLT3819H‐V, Shawnee, KS, USA). End tidal partial pressures of O2 (PETO_2_: mmHg) and CO_2_ (PETCO_2_: mmHg) were calculated from fraction of expired oxygen (FeO_2_; %) and carbon dioxide (FeCO_2_; %) via gas analyzer (ADI gas analyzer, ML206, Colorado Springs, CO, USA) and daily atmospheric pressure. End tidal partial pressures were corrected for body temperature and pressure, saturated water vapor (BTPS; [Torr]). Supplemental gases (see below) were provided as required via a 6 Liter rebreathing bag attached to a 3‐way valve (Hans Rudolph Inc., 2870, Shawnee, KS, USA). This was required for the rebreathing protocol, end‐inspiratory breath‐hold and end‐expiratory breath‐hold (See below). Two separate gas mixtures (FiO2 1.00, and previously collected expired air (i.e., subject specific FeO_2_ ~0.15; and FeCO_2_ ~0.05) were administered during these protocols. A 3‐way valve (Hans Rudolph Inc., 2870, Shawnee, KS, USA) was implemented to switch between room air (FiO_2_ 0.2093; Pb 701 ± 3 Torr) and each respective gas mixture.

Microneurography was used to measure muscle sympathetic nerve activity (MSNA) (Hagbarth and Vallbo 1968; Steinback et al. 2010a). A recording electrode (200 *μ*m in diameter, 35 mm long with a tapered uninsulated tip 1–5 *μ*m) was placed percutaneous into the right peroneal while the reference electrode was positioned approximately 1–3 cm away from the recording site. The recording electrode was manually manipulated after insertion until a characteristic pulse‐synchronous burst pattern was obtained. Confirmation of MSNA occurred when apnea produced pulse synchronous bursts and an absence of skin parasthesisa was observed (Delius et al. 1972). The raw MSNA signal was amplified 1000× initially through a preamplifier and then 100× through a variable gain isolated amplifier. The raw signal was band pass filtered (700–2000 Hz), rectified, and integrated (decay constant 0.1 sec) to obtain a mean voltage neurogram (model 662C‐3; Iowa University Bioengineering. Both raw and integrated signals were sampled at 10,000 Hz and 1000 Hz respectively and stored for offline analysis (ADInstruments, Chart Pro v8.3.1).

### Experimental protocols

**Figure 1:**
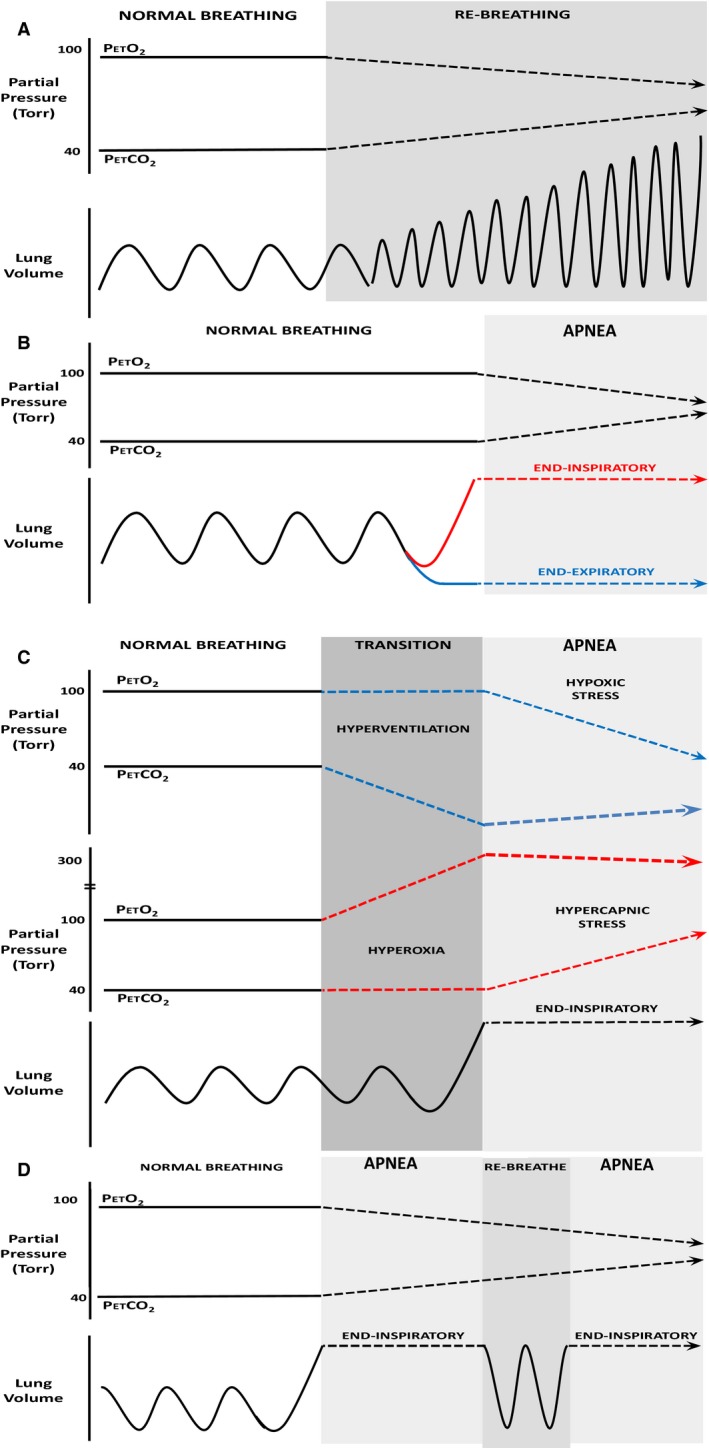
Visual depiction of different maneuvers performed during each specific protocol. (A) Rebreathe protocol where individuals breathe from a bag containing their expired tidal gases. Participants performed the rebreathe protocol until the specified cut‐off criteria was reached (PETO_2_ <45 mmHg or PETCO_2_ >60 mmHg). (B) Participants performed end two breath‐holds at either total lung capacity (End‐Inspiratory) or at functional residual capacity (End‐Expiratory). (C) *Participants* were given either supplemental oxygen (FiO_2_ 1.00, [HYP]) or instructed to hyperventilate (PETCO_2_ <25 mmHg, [HV]) prior to commencing a maximal inspiratory breath‐hold. (D) Participants performed a repeated rebreathe protocol. Following the volitional breakpoint of the first breath‐hold, individuals were allowed 2–3 breaths from a bag containing their respective end‐tidal gases. Following these breaths they performed another maximal inspiratory breath‐hold. This was done for three maximal inspiratory breath‐holds. All maneuvers were followed by a 10‐min washout period prior to establishing the following baseline in order to allow end tidal values to return to normal. Participants were instructed and encouraged to hold their breath for “as long as possible”.

Following instrumentation and a baseline period of 10 min, participants performed a randomized series of asphyxic rebreathe and maximal BH trials (Fig. 1). Each trial was separated by a minimum 10‐min washout period that allowed for PETO_2_, PETCO_2_ and MSNA values to return to baseline levels.

### Maximal breath‐hold trials

Four maximal BH trials were performed in random order to isolate specific mechanical/chemical reflexes: (1) End Inspiratory BH (INS); (2) End Expiratory BH (EXP); (3) Prehyperventilation maximal BH (HV); (4) Prehyperoxia maximal BH (HYP). Pulmonary stretch was assessed through the performance of two separate maximal BH maneuvers performed at end‐Inspiratory volume (INS) or at functional residual capacity (EXP). All participants performed BHs and rebreathing maneuvers with a facemask attached in order to obtain end‐tidal values. Each BH was kept within the range of tidal volume to reduce sympathetic potentiation through excessive baroreflex (un) loading, as seen previously with large lung volumes (Heusser et al. 2009). Central and peripheral chemoreflex pathways were assessed through two maneuvers eliciting hypercapnic (HYP) and hypoxic (HV) stimulus. Central chemoreflex activation was isolated via supplemental oxygen (FiO_2_ 1.00 for 22 ± 11 sec) until PETO_2_ plateaued (>300 Torr) that was meant to inhibit peripheral chemoreceptor activation during BHs (Eyzaguirre and Lewin 1961). This was immediately followed by the performance of a maximal inspiratory BH. The peripheral chemoreflex was isolated through hyperventilation to reduce PETCO_2_ (<25 Torr over 90 ± 19 sec) prior to maximal inspiratory BH. Subjects were instructed to hold their breath for “as long as possible” during each protocol until volitional breakpoint. Upon reaching breakpoint, participants were coached through 2–3 successive breaths from a rebreathing circuit that allowed for collection of final PETO_2_ and PETCO_2_ values. Both BH duration and the volitional breakpoint of each subject were confirmed through cessation and return of respiratory flow.

### Rebreathe trial

The influence of dynamic ventilation during heightened chemoreflex stress on MSNA was assessed through the performance of a rebreathe trial (RB). The prebaseline period consisted of collecting participant's expired gases during normal tidal volume breathing via a 6L rebreathing bag. Expired gases were collected for several seconds until the bag was full. They were then allowed a 5‐min baseline period breathing room air prior to switching a 3‐way valve over to the bag containing expired gases. Participants were instructed to breathe normally following the switch to the bag until: (1) A designated cutoff point was reached (PETO_2_ = 45 Torr or PETCO_2_ = 65 Torr; sampled from the rebreathing circuit); (2) the participant signaled for the protocol to be stopped; or (3) the rebreathing bag was emptied. Participants were then switched back to room air and allowed to return back to baseline values.

### Repeated rebreathe trial

The interactive effects of progressive chemoreflex stress during successive BHs were assessed through a series of repeated rebreathing BH maneuvers (RRB). This was the same technique used by Steinback et al. (2010a) and adapted from the protocol originally reported by Fowler (1954). After the initiation of an inspiratory BH participants were switched over from room air to an empty 6L rebreathing bag. They were instructed to “hold their breath for as long as possible” until volitional breakpoint. Following breakpoint, participants were coached to exhale and subsequently take two normal breaths from the rebreathing apparatus, followed by a subsequent maximal inspiratory BH. This was repeated for at least 3 BH trials (RRB 1, RRB 2, and RRB 3) before being switched back to room air. PETO_2_ and PETCO_2_ were collected during the two‐normal breaths between each trial, and following the last trial, to determine the progressive change in chemoreflex stimulation (see below).

### Data collection and analysis

MSNA and cardiovascular data (TPR, CO, HR, MAP) were analyzed during the last minute of baseline and 15 sec prior to volitional breakpoint. Maximal BH durations and volitional breakpoint were determined through analysis of spirometry recordings to the nearest second. Bursts were identified and confirmed by a trained observer (SAB/CDS). Integrated MSNA signals were quantified as: burst frequency (bursts/min), burst incidence (bursts/100 heart beats [hb]), burst amplitude (% increase normalized to peak baseline burst amplitude), and total MSNA activity (burst frequency*normalized amplitude; au). Peak MSNA data were collected during the last 15 sec of each breath‐hold in order to observe the maximal MSNA response. The last 15 sec of MSNA was also collected during the rebreathing breath‐holds. RRB trials that were shorter than 15 sec had averages calculated for the entire duration of the breath‐hold in question.

**Figure 2:**
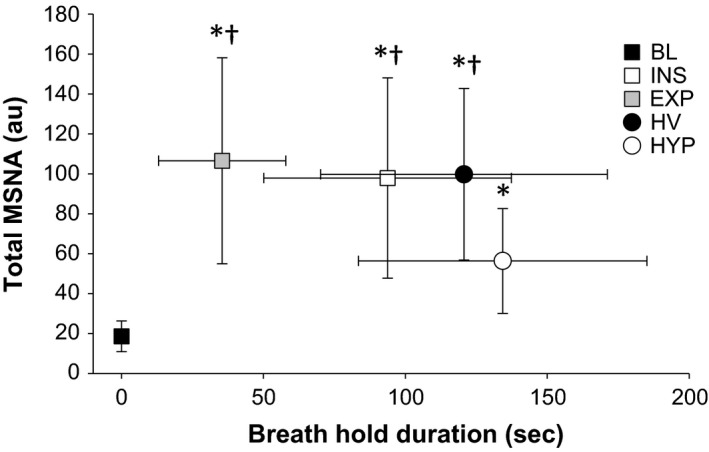
Scatterplot representation of maximal breath‐holds total MSNA (au) and breath‐hold durations (sec) for baseline (BL), End‐Inspiratory (INS), End‐Expiratory (EXP), Pre‐Hyperventilation (HV), and Pre‐Hyperoxia (HYP) protocols. BL duration represented with “O seconds”, as no breath‐holding was performed. All breath‐hold protocols saw a significant increase in Total MSNA (*P* < 0.01) prior to volitional breakpoint. The INS, EXP, and HV protocol saw significantly larger Total MSNA versus HYP protocol prior to volitional breakpoint, despite HYP duration being the longest. *Significant difference versus baseline (*P* < 0.01); ^†^Significant difference versus HYP protocol (*P* < 0.01).

Combined chemoreflex stress during rebreathing and INS was calculated using a previously defined “stimulus index”(Bruce et al. 2016). Briefly, this index assesses an increase in chemoreflex activation by assuming that changes in PETO_2_ and PETCO_2_ during breath‐holding are linear within healthy individuals that exhibit normal metabolic function. Therefore, a greater stimulus index is indicative of a larger degree of combined asphyxic strain during breath‐holding. The linear change in end‐tidal values was interpolated on a breath‐by‐breath basis immediately prior to commencement of each BH, and terminated following volitional breakpoint. Stimulus index was determined across all RB and BH trials. In addition, a stimulus index “Isopoint” was calculated between the RB and INS Breath‐Hold trial (Fig. 2). Both the RB (measured) and INS (interpolated) stimulus index values were aligned and compared, with MSNA values averaged for 15 sec around the Isopoint.

### Statistical analysis

All measurements of MSNA were compared baseline values (Table 1). Results are reported as mean ± standard deviation. Two separate comparisons were performed utilizing one‐way repeated measures ANOVA (Systat Software, San Jose, CA) were conducted (*P* < 0.05). The first comparisons were between baseline, INS, EXP, HV, and HYP trials. The second was between BL, and RRB1, RRB2, and RRB3 trials. A Holm–Sidak Test correction (*P* = 0.05) was used as a Post‐Hoc analysis for one‐way ANOVAs that came up significant. Mann–Whiney tests were run in incidences of non‐normal distributions. MSNA obtained at aligned Stimulus Index iso‐points from the RB and INS trials was compared through student's paired *T*‐test (*P* = 0.05).

**Table 1:** Subject demographic, MSNA, spirometry, and cardiovascular measures. Data represented as mean ± standard deviation

	N. 10 (*F* = 6)
Demographics	
Age (years)	24 ± 3
Height (m)	1.71 ± 0.13
Weight (Kg)	69 ± 11
Basal MSNA	
Burst frequency (bursts/min)	21 ± 11
Burst incidence (bursts/100 hb)	26 ± 11
Total MSNA (au)	19 ± 8
Spirometry	
PETO_2_ (Torr)	105 ± 4
PETCO_2_ (Torr)	44 ± 2
Stimulus index (au)	0.41± 0.03
FVC (L)	4.8 ± 1.0
RV (L)	1.3 ± 0.3
TLC (L)	6.0 ± 1.3
Cardiovascular	
SpO_2_ (%)	98 ± 1
Heart rate (bpm)	69 ± 25
♦Cardiac output (L/min)	6.8 ± 4.2
Mean arterial pressure (mmHg)	89 ± 31
Systolic blood pressure (mmHg)	124 ± 45
Diastolic blood pressure (mmHg)	67 ± 23
♦Total peripheral resistance (L/min/mmHg)	13.1 ± 5.5

♦Model flow algorithm (Finometer Pro, Finapres Medical Systems, Netherlands).

## Results

Participant demographics, resting cardiovascular, respiratory, and sympathetic characteristics are outlined in Table 1.

### Breath‐hold trials

Baseline and BH measures are listed in Tables 1 and 2. Average BH times varied depending on maneuver (range 29–134 sec) with HYP yielding the longest BH duration (134 ± 48 sec). Prior to volitional breakpoint all maximal BH trials resulted in reductions in PETO_2_ and increases in PETCO_2_. The increase in SI was largest within the INS trial (+0.26 ± 17 au from baseline; *P* < 0.05), though SpO_s_ was lowest within the HV BH (91 ± 4). MAP increased during all trials (*P* < 0.01), while TPR was elevated in HV and HYP conditions (*P* < 0.01) but not RB (Table 2). There was no difference in HR responses between maneuvers.

**Table 2:** Summary of rebreathe and breath‐hold times and changes in respiratory and cardiovascular measures during trials. Data represented as mean ± standard deviation

	RB	INS	EXP	HV	HYP
Breath‐hold time (Sec)	165 ± 31	68 ± 21^b^, ^c^, ^d^	29 ± 15^b^, ^c^, ^d^	124 ± 38^b^	134 ± 48^b^
Stimulus index (au)					
Δ PETO_2_ (Torr)	−54 ± 13^a^	−26 ± 11^a^, ^b^, ^c^, ^d^	−16 ± 17^a^, ^b^, ^c^	−60 ± 16^a^, ^c^	+312 ± 64^a^, ^b^
Δ PETCO_2_ (Torr)	+13 ± 7^a^	+10 ± 3^a^, ^b^, ^c^, ^d^	+4 ± 5^b^, ^c^	+3 ± 4^b^, ^c^	+12 ± 5^a^
Δ Stimulus index (au)	+0.57 ± 0.27^a^	+0.26 ± 0.17^a^, ^b^, ^c^, ^d^	+0.16 ± 0.14^a^, ^b^, ^c^	+0.24 ± 0.18^a^, ^b^, ^c^	−0.12 ± 0.10^a^, ^b^
Cardiovascular					
SpO_2_ (%)	84 ± 6^a^	93 ± 3^a^, ^b^, ^c^	95 ± 2^b^, ^d^	91 ± 4^a^, ^b^, ^c^	99 ± 1^b^, ^d^
Δ Heart rate (bpm)	+16 ± 13^a^	−7 ± 15^b^	−8 ± 12^b^	0 ± 15^b^	−4 ± 19^b^
Δ Mean arterial pressure (mmHg)	+20 ± 9^a^	+24 ± 9^a^	+24 ± 7^a^	+21 ± 5^a^	+21 ± 8^a^
Δ Systolic blood pressure (mmHg)	+26 ± 17^a^	+23 ± 9^a^	+21 ± 8^a^	+23 ± 14^a^	+20 ± 6^a^
Δ Diastolic blood pressure (mmHg)	+17 ± 7^a^	+23 ± 9^a^	+21 ± 8^a^	+19 ± 3^a^	+20 ± 6^a^
♦Δ Cardiac output (L/min)	+1.0 ± 0.9	−1.4 ± 1.6 b	−1.0 ± 1.6	−0.7 ± 1.3^b^	−0.9 ± 1.4^b^
♦Δ Total peripheral resistance (mmHg/L/min)	+8.2 ± 15.6	+9.4 ± 9.0	+6.5 ± 4.8	+5.0 ± 3.1^a^, ^b^	5.1 ± 3.0^a^

RB, Rebreathing Trial; INS, Inspiratory breath‐hold trial; EXP, Expiratory breath‐hold trial; HV, Prehyperventilation breath‐hold trial; HYP, Prehyperoxia (FIO_2_ = 1.00) breath‐hold trial. RB Breath Hold Duration represents the period participants were rebreathing prior to recovery.

♦ Model Flow.

a Significant difference compared to Baseline (*P* < 0.01).

b Significant difference compared to rb (*P* < 0.01).

c Significant difference compared to HYP (*P* < 0.01).

d Significant difference compared to HV (*P* < 0.01).

**Figure 3:**
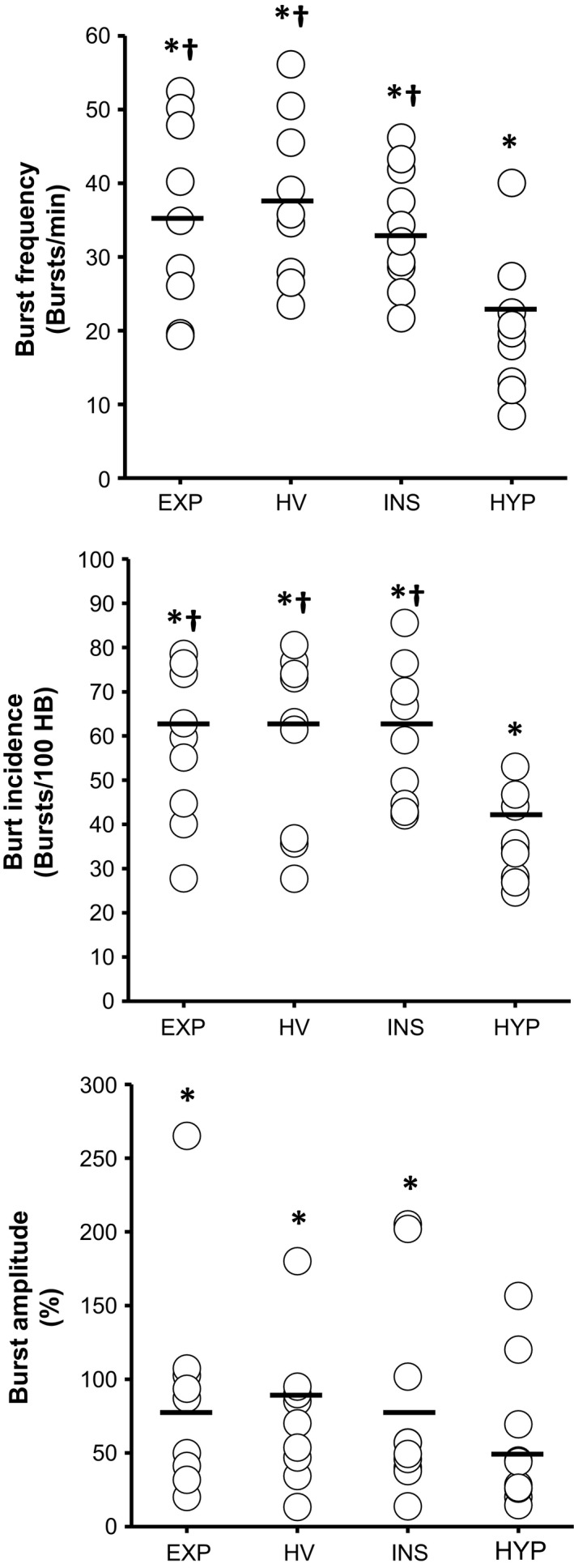
MSNA responses during INS, EXP, HV, and HYP protocols. Values represent increase respective of baseline for burst frequency (burst/min), burst incidence (burst/100 hb), and burst amplitude (%). Burst frequency and incidence was increased across all protocols (*P* < 0.01). Burst amplitude was increased for INS, EXP, and HV protocols only (*P* < 0.01). Burst frequency and incidence, and amplitude was similar between INS, EXP, and HV protocols. Burst frequency and incidence was lower in HYP protocol prior to volitional breakpoint. *Significant difference versus baseline (*P* < 0.01); ^†^Significant difference versus HYP protocol (*P* < 0.01).

MSNA values for the four BH trials are represented in Figures 2 and 3. Total MSNA was increased during all BH trials prior to volitional breakpoint (BL 19 ± 8; INS 98 ± 50; EXP 107 ± 52; HV 100 ± 43; HYP 56 ± 26 au; *P* < 0.01). This included an increase in burst frequency (INS 53 ± 11; EXP 55 ± 13; HV 56 ± 14; HYP 40 ± 12 burst/min; *P* < 0.001), and burst incidence (INS 77 ± 20; EXP 77 ± 20; HV 77 ± 25; HYP 54 ± 15 burst/100 hb; *P* < 0.001). Burst amplitude was only significantly elevated in the INS (+81 ± 68%), EXP (+89 ± 74%) and HV (+74 ± 48%) trials (*P* < 0.01). However, the increase in MSNA (Total, burst frequency, and burst incidence) was lower in HYP respective of the other breath‐hold trials (*P* < 0.01).

### Isopoint and MSNA

**Figure 4:**
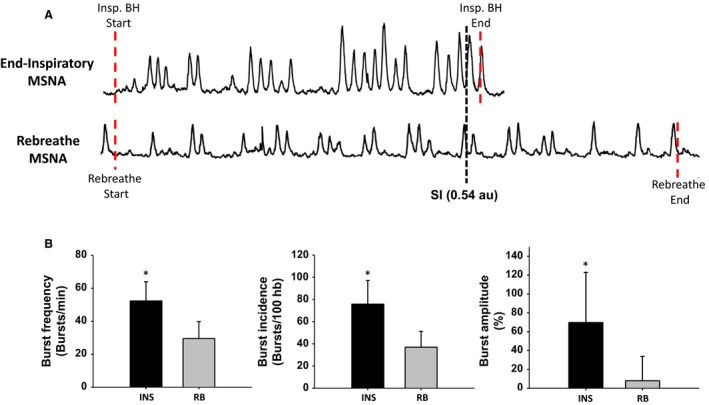
Schematic and results obtained from iso‐point data obtained during rebreathe (RB) and end inspiratory (INS) protocol. (A) Iso‐point was selected through the calculation of a stimulus index (ratio of end tidal CO_2_ and O_2_ [au]) and matched at specific time points within each participant (S.I. 0.54). End‐tidal was determined during the INS protocol through an extrapolated linear slope based off the change in end tidal values (pre to post breath‐hold). (B) Burst frequency (bursts/min), burst incidence (burst/100hb), and burst amplitude (% increase vs. baseline) obtained from the RB and INS isopoints. Burst frequency, incidence, and amplitude was larger in the INS protocol compared to RB protocol for their respective chemoreflex stimulus. *Significant difference versus rebreathe protocol at that respective iso‐point (*P* < 0.01).

RB trials (presented in Table 2) were the longest trials for all participants (182 ± 72 sec). RB also demonstrated the biggest SI (0.98 ± 0.27 au), with an overall reduction in PETO_2_ (−46 ± 11 Torr) and increase in PETCO_2_ (13 ± 7 Torr). Only the HYP trial demonstrating a similar PETCO_2_ (12 ± 5 Torr). SpO_2_ was also the lowest within the rebreathe protocol compared to all other breath‐hold maneuvers (*P* < 0.05). Unlike the BH trials, RB saw an increase in HR above baseline (*P* = 0.036) alongside increases in MAP (*P* = 0.004), SBP (*P* = 0.027), DBP (*P* < 0.001). MSNA was increased as well, with an elevated burst frequency and incidence compared to baseline (*P* < 0.01), but not burst amplitude (*P* = 0.274). The Isopoint for RB and INS trials are shown in Figure 4. The stimulus index (0.54 au) between RB and INS trials were matched for similar PETO_2_ (80 Torr) and PETCO_2_ (51 Torr) values. At the same stimulus index, overall MSNA was lower during the RB maneuver. This was seen with a lower burst frequency (RB 30 ± 10 vs. INS 52 ± 12 bursts/min; *P* < 0.01), burst incidence (RB 39 ± 12 vs. INS 76 ± 21 bursts/100 hb; *P* < 0.01), and % increase in burst amplitude (RB +8 ± 26% vs. INS +70 ± 53%; *P* < 0.01).

### Repeated rebreathe trials

**Figure 5:**
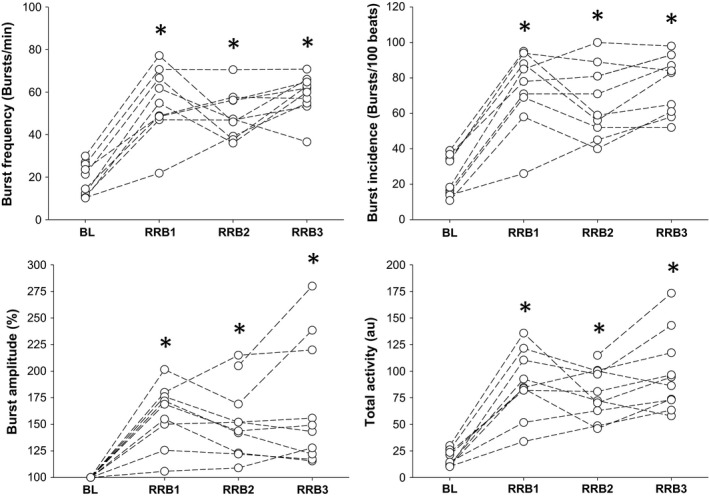
MSNA measures during baseline (BL) and breath‐holds 1, 2, and 3 of the repeated rebreathe protocol (RRB1, RRB2, RRB3, respectively). MSNA represented as burst frequency (burst/min), burst incidence (burst/100 hb), burst amplitude (%), and Total MSNA (au). Burst frequency, incidence, amplitude, and total MSNA was increased across all conditions receptive of baseline. However, MSNA was similar in each breath‐hold, despite greater chemoreflex stimulus and shorter breath‐hold duration. *Significant difference versus rebreathe protocol at that respective iso‐point (*P* < 0.01).

Overall BH times, cardiovascular, and stimulus index for each successive RRB BH are listed in Table 3. All participants were able to complete at least three successive RRB trials. BH trial durations became shorter with each successive trial (RRB1 71 ± 22 vs. RRB2 29 ± 9 vs. RRB3 19 ± 6 sec; Condition Main Effect *P* < 0.01), while SI increased (RRB1 +0.25 ± 0.13 vs. RRB2 +0.42 ± 0.18 vs. RRB3 +0.56 ± 0.25 au; *P* < 0.01). Though SpO_2_ was not obtained during RRB 1 and RRB2, the nadir response post RRB 3 volitional breakpoint reached 86 ± 9% (*P* < 0.01 vs. baseline). TPR was significantly elevated for RRB1 and RRB3 (*P* < 0.01). MSNA response is represented in Figure 5. Compared to baseline, Total MSNA was higher (*P* < 0.01) across all 3 trials (BL 19 ± 8 vs. RRB1 90 ± 31 vs. RRB2 79 ±  23 vs. RRB3 94 ± 44 au). This included an increase in burst frequency (*P* < 0.01), burst incidence (*P* < 0.01), and burst amplitude (*P* < 0.01). However, burst frequency, incidence, and amplitude were similar across breath‐holds, despite shorter breath‐hold duration, and larger stimulus index, with each successive trial.

**Table 3:** Summary of breath‐hold times and cardiovascular measures obtained during repeated rebreathe trials. Data represented as mean  ±  standard deviation

	RRB 1	RRB 2	RRB 3
Breath‐hold time (Sec)	71 ± 22	29 ± 7^b^	19 ± 6^b^
Stimulus index (au)			
Δ PETO_2_ (Torr)	−28 ± 12^a^	−38 ± 15^a^	−44 ± 15^a^, ^b^
Δ PETCO_2_ (Torr)	+7 ± 5^a^	+11 ± 4^a^, ^b^	+13 ± 4^a^, ^b^, ^c^
Δ Stimulus index (au)	+0.25 ± 0.13^a^	+0.42 ± 0.18^a^, ^b^	+0.56 ± 0.25^a^, ^b^, ^c^
Cardiovascular			
SpO_2_ (%)	–	–	86 ± 9^a^
Δ Heart rate (bpm)	−5 ± 9	−4 ± 11	−2 ± 8
Δ Mean arterial pressure (mmHg)	+17 ± 5^a^	+13 ± 7^a^	+15 ± 9^a^
Δ Systolic blood pressure (mmHg)	+21 ± 15^a^	+20 ± 13^a^	+25 ± 17^a^
Δ Diastolic blood pressure (mmHg)	+17 ± 5^a^	+13 ± 7^a^	+15 ± 9^a^
♦Δ Cardiac output (L/min)	−1.2 ± 1.1^a^	−0.7 ± 0.8	−0.5 ± 1.1
♦Δ Total peripheral resistance (mmHg/L/min)	+7.2 ± 3.4^a^	+4.7 ± 3.4	+5.2 ± 5.6^a^

NOTE: SpO_2_ not obtained during Repeated Rebreathe BH trial 1 (RRB 1) and trial 2 (RRB 2) due to delay associated in reading values being longer than the interval between breath‐holds. This would not allow for an accurate reading of the nadir response following each trial. The SpO_2_ during Repeated Rebreathe BH trial 3 (RRB 3) was obtained from the nadir response within 30–40 sec post volitional breakpoint.

^♦^ Model Flow.

a Significant difference compared to Baseline (*P* < 0.01).

b Significant difference compared to RRB 1 (*P* < 0.01).

c Significant difference compared to RRB 2 (*P* < 0.01).

## Discussion

The current study delineates the specific mechanisms that contribute to the peak MSNA response during several maximal BH maneuvers. Our results indicate four main findings: (1) The peripheral chemoreflex plays an obligatory role in the sympathetic response prior during a maximal BH attempt. This was based off the findings that supplemental oxygen prior to our maximal INS BH resulted in a consistently smaller increase of MSNA. (2) The overall maximal MSNA response does not appear to be affected by the initial pre‐BH lung volume. (3) voluntary BH resulted in an apparent maximal sympathetic response immediately prior to volitional breakpoint within the INS, EXP, HV, and RRB maneuvers, despite varying BH duration and states of asphyxic strain. (4) Dynamic ventilation has a suppressive effect on sympathetic outflow when chemoreflex stress is matched between conditions.

### The chemoreflex role in MSNA augmentation during volitional breath‐holding

One of the main finding within our study was the significantly lower MSNA response during the HYP BH compared with the HV BH, despite a similar BH duration between maneuvers. This confirms previous findings by Hardy et al. (1994) and Steinback et al. (2010a). while further demonstrating the necessity of the peripheral chemoreceptors in producing a peak sympathetic response prior to volitional breakpoint. However, it must also be noted that there does not appear to be a graded response by which heightened peripheral chemoreceptor discharge contributes to further increases in MSNA. Though greater chemoreceptor afferent activity occurs under progressive reductions in arterial oxygen tension (Marshall 1994; Kumar and Prabhakar 2012), there is only a small degree of discharging until an arterial oxygen content threshold of 60 mmHg is reached (Hornbein et al. 1961; Vidruk et al. 2001). It is beyond that point where a exponential increase in discharge frequency occurs at progressively lower PaO_2_. This is relevant given that our HV trial reached a similar maximal MSNA response (compared to the INS and EXP BH) while only reaching a PETO_2_ of 73 Torr (SpO_2_ nadir of 86 ± 9%). We also noted a larger reduction in end tidal O_2_ during the INS trial with no difference in MSNA compared to the EXP trial (which had smaller hypoxic stress). Therefore, the activation of the peripheral chemoceptors is necessary in generating a peak MSNA response, though greater hypoxic strain does not appear to translate towards further sympathetic augmentation as seen by the similar MSNA responses between INS and HV protocols.

The lack of difference within the HV BH respective to the INS and EXP trials argues that the central chemoreflex does not contribute towards this peak MSNA response. This is not surprising and may be explained through the PCO_2_ gradient between the cerebrospinal fluid and cerebral arteries. Despite the central chemoreceptors being more responsive to small changes in blood brain pH through metabolic PCO_2_ production and PaCO_2_ accumulation (Pappenheimer et al. 1965; Ainslie and Duffin 2009), the time required for medullary PCO_2_ concentrations to match that of PaCO_2_ has been estimated to require as long as 5 min (Farhi and Rahn 1960; Ainslie and Duffin 2009). In addition, the inverse relationship between medullary CO_2_ concentrations is tightly regulated through an inverse relationship with cerebral perfusion in a negative feedback loop, where increased medullary CO_2_ activation also undergoes increased cerebral flow (Nattie and Li 2012; Ainslie et al. 2013). Indeed within free breathing models where hypoxic and hypercapnic stresses are isolated during longer exposure periods, hypercapnia has shown considerably higher MSNA potentiation (Somers et al. 1989a; Jouett et al. 2015). Therefore, the lack of change in MSNA during the HV protocol may be simply attributed to BHs not being long enough to evoke true activation of the central chemoreflex. However, we acknowledge that there may be an effect of heightened central (hypercapnic) chemoreflex drive on peak MSNA within extreme BHs prior to volitional breakpoint, such as in elite BH divers (Somers et al. 1989a; Steinback et al. 2009, 2010a).

An interesting finding within the RRB BH trials was that the peak MSNA responses were similar. The repeated rebreathe model allowed for assessment of increased asphyxic stress under voluntary apnea, with each successive BH becoming progressively shorter while chemoreflex activation gradually increased. Without chemical relief (ie. return to normoxic conditions) following the volitional breakpoint the heightened afferent chemoreceptor activation and theoretically result in greater sympathetic potentiation during long‐term BHs (Somers et al. 1989a; Hardy et al. 1994; Heusser et al. 2009). As such, we initially hypothesized that this accumulated asphyxic strain would exert further MSNA potentiation with each successive BH. Though our findings of a successively shorter BH duration agrees with Fowler (1954), alongside a more rapid onset of MSNA potentiation following return to apnea; the peak MSNA response was similar between the three trials. The concept of a potential sympathetic ceiling has been suggested previously using models that utilize excessive baroreflex unloading/activation (Cooke et al. 2009; Fagius and Nygren 2010), though previous accounts involving significant chemoreflex activation (Somers et al. 1989a) have also produced no further increases of MSNA. However, Steinback et al. (2010b)) did not find the presence of a sympathetic ceiling during several successive BHs while inspiring their captured expired gases between BHs, though their observed MSNA responses were notably lower than what was observed within this study, in addition to a lesser nadir in oxygen desaturation. Though not directly measured within our study, we propose that this ceiling is potentially limited through either the efferent arm, or sensory related through a potential finite ability for afferent chemoreceptor activation to occur (Somers et al. 1989b). Our findings saw both burst frequency and incidence were maximized during the three RRB trials, limiting further increases in MSNA solely to burst amplitude. However, the normalized amplitudes were similar between the three trials, despite shorter breath‐hold duration and a larger chemical drive. Thus, we conclude from these RRB trials that a sympathetic ceiling may exist, where no further increases in total MSNA occur despite greater chemoreflex activation.

### The peak MSNA during breath‐holding is not affected by initial lung‐volume

In the current study we have demonstrated that the peak MSNA response was similar between the INS and EXP BH trials despite BH duration varying between each maneuver (range). In regards to the similar peak responses between the INS and EXP BHs, initial static lung volume does not appear to play a role. This is interesting given BHs at a higher lung volume will prolong BH duration through recruitment of inhibitory afferent pulmonary/lung stretch receptors that mitigate the ventilatory drive‐to‐breathe (Macefield and Wallin 1995b; Parkes 2006; Bain et al. 2018). As both the ventilatory drive and efferent sympathetic outflow pathways share similar pathways within the central neuronal pools of the medulla (Guyenet 2000), we hypothesized that the slope of MSNA potentiation (and ultimately peak response) within our EXP maneuver would differ to that INS through unloading such pulmonary/lung wall receptors. However, it appears that INSP and EXP resulted in similar peak sympathetic responses, despite different BH durations. The lack of pulmonary afferents contribution to the peak MSNA response agrees with earlier finding by Khayat et al. (2004) that found no difference in the overall MSNA response to maximal apnea between lung‐denervated patients and controls during a maximal EXP BH maneuver. Therefore initial lung volume although affects BH duration, but does not promote a sympatho‐inhibitory effect prebreakpoint.

Though our findings suggest that the peak MSNA response is not affected by the pulmonary stretch reflex, we acknowledge that initial BH lung volume can indirectly alter MSNA augmentation earlier on during apnea. Previous studies demonstrate a biphasic response in MSNA, where the initial BH period is governed through an interaction between pulmonary/lung stretch activation and baroreflex unloading (Macefield and Wallin 1995b; Macefield et al. 2006; Heusser et al. 2009; Breskovic et al. 2011). It has been proposed that higher initial BH lung volumes see an earlier elevation of MSNA to counteract increased intrathoracic pressures (Macefield and Wallin 1995a). Heusser et al. (2009) previously reported an initial surge in sympathetic drive concurrent with overall MAP reduction within 15‐20 sec of maximal inspiration breath‐holding. They later demonstrated additional MSNA augmentation under excessive loading within the lungs, such as lung packing seen in breath‐hold divers (Heusser et al. 2010). However, an initial spike in MSNA does appear at lower lung volumes through an assumed reduction pulmonary stretch feedback, rather than baroreflex‐unloading (Macefield and Wallin 1995b). Greater lung volume would concurrently increase baroreflex and pulmonary stretch reflexes, although the interaction between them is less understood.

### MSNA response to dynamic ventilation versus static lung volume and sympathetic activity

The rebreathing protocol aimed to evaluate the effects of chemoreflex activation (hypercapnic and hypoxic) while simultaneously removing respiratory related feedback from pulmonary/lung stretch receptors. We have previously reported similar findings that show lower sympathetic augmentation with rebreathing compared to an end‐expiratory BH (Steinback et al. 2010b). However, the former study did not show the influence of dynamic ventilation on MSNA at two matched chemical stressors. More recently, Badrov et al. (2017) also demonstrated lower action potential recruitment when they matched a similar rebreathing protocol to maximal BHs at functional residual capacity within elite BH divers. Within our own findings, when stress is matched between rebreathing and our BH (end‐inspiratory), rebreathing exhibited a markedly blunted MSNA response. Our findings further demonstrate that dynamic ventilation has a strong suppressive effect of sympathetic outflow under chemoreflex activation. Pulmonary stretch discharge follows a cyclical pattern during normal ventilation, with increased firing during the inspiratory phase followed by cessation during expiration (Somers et al. 1989b; Seals et al. 1990, 1993) meant to prevent lung over‐inflation. Sympathetic outflow also follows a similar pattern of respiratory modulation, with increased pulmonary stretch feedback during late inspiration suppressing the MSNA response under normal ventilation(Hagbarth and Vallbo 1968; Eckberg et al. 1985; Seals et al. 1990; Macefield and Wallin 1995a) and during artificial ventilation within lung‐denervated patients(Seals et al. 1993). However, sympathetic outflow still remains tightly regulated to the respiratory cycle such that MSNA is suppressed during the inspiratory phase (Somers et al. 1989a; Seals et al. 1990; Jouett et al. 2015). Thus, these findings agree with other accounts (Somers et al. 1989b; Steinback et al. 2010b) that demonstrate ventilatory restraint of sympathetic activity should be an important consideration for future free breathing chemoreflex studies, which may not be for the pulmonary withdrawal of the true MSNA to greater asphyxic strain.

### Considerations & limitations

It is important to acknowledge within the current study that these maximal BH MSNA responses were observed within untrained individuals that had no previous history of competitive breath‐holding, competitive swimming, or diving. Average BH durations vary greatly between untrained and trained individuals. For example, elite divers are capable of holding their breath for exceptionally long periods of time that are well beyond the onset of involuntary breathing movements and often induce volitional breakpoints within untrained individuals(Andersson and Schagatay 1998; Dujic et al. 2008; Heusser et al. 2009; Bain et al. 2017). This is important given that Heusser et al. (2009)) previously demonstrated during maximal static BHs, elite BH divers are capable of achieving an overall lower arterial oxygen saturation, longer BH duration, and greater total MSNA response when compared with untrained individuals. What is interesting within these findings is that this similar group of elite divers exhibits normal peripheral and central chemoreflex sensitivity (Grassi et al. 1994; Dujic et al. 2008; Steinback et al. 2010a) action potential recruitment patterns during maximal BH attempts (Breskovic et al. 2011) compared to untrained individuals. Therefore, an individual's maximal MSNA response may be dictated by their ability to tolerate significant chemoreflex strain, with longer BH durations promoting larger maximal MSNA responses. Within this study, the BH trial that exhibited an attenuated increase in MSNA (the HYP trial) response also had the lowest chemoreflex strain (SI = −0.12). However, the similar MSNA responses within our untrained participants RRB trials, despite larger degrees of chemoreflex strain and shorter BH durations, disagree with the lack of MSNA ceiling previously seen (Heusser et al. 2009). Furthermore, our lowest SpO_2_ achieved in any of the breath‐holds does not match what was seen previously within other studies utilizing elite BH divers (Heusser et al. 2009; Breskovic et al. 2011). Although further investigation is required, the findings of our current study argue that there is a finite amount of postganglionic sympathetic neuron recruitment that can be achieved until additional chemoreflex activation sees no further increases in MSNA.

Volitional breath‐holding is a complex interaction between several reflexes that alter both the ventilatory and sympathetic pathways within the brainstem. As such, there lies other potential underlying mechanisms that we did not investigate which may contribute to these findings. One example is that breaths hold tolerance involves a significant mental component to counteract any urge to breathe. Breath‐hold duration can be prolonged by previous repetitive practice (Parkes 2006), trained versus untrained individuals (Heusser et al. 2009; Breskovic et al. 2011), and mental distractions (Alpher et al. 1986). As there is arguably considerable cognitive stress involved with breath holding, this also needs to be acknowledged within the current study. However, we attempted to address the effect of repetitive practice on breath through randomization of the breath‐hold maneuvers. This would reduce the effects that a nonrandomized breath‐hold protocol would have on MSNA and breath‐hold tolerance. Another mechanism possibly at play is baroreflex interaction as blood pressure progressively rose during each of our trial prior to volitional breakpoint. With a rise in arterial pressure there would be the potential for inhibitory feedback to central cardiovascular control centers due to increased cardiac and carotid baroreflex mediated activation. As such we cannot confirm that the current findings of sympathetic augmentation may be due partially to baroreflex withdrawal.

## Conclusion

There is considerable sympathetic augmentation under maximal volitional breath‐holding. However, we have demonstrated though several unique BH maneuvers that the peripheral chemoreceptors play a considerable role in this sympathetic response. In particular, the peripheral chemoreflex may be essential for maximizing the potentiation of sympathetic activity prior to volitional breakpoint, though it does not necessarily translate to a larger sympathetic response with even greater hypoxic stress. Though breath‐hold duration varies based on initial lung volume, the effect of pulmonary stretch does not appear to alter the maximal MSNA response prior to volitional breakpoint. Yet when similar chemoreflex stresses are matched between dynamic ventilation and static lung volume; sympathetic activation is lower during ventilation. Finally, the evidence of a potential sympathetic plateau within several of our maneuvers is interesting and demonstrates the considerable sympathetic stressor that underlies the performance of maximal breath‐holds.

## Conflict of Interest

None declared.
